# Supplementary figures and images for: Highly Reproducible 16S Sequencing Facilitates Measurement of Host Genetic Influences on the Stickleback Gut Microbiome
Source: mSystems. 2019 Aug 13;4(4):e00331-19. doi: 10.1128/mSystems.00331-19 (PMC6697441; doi:10.1128/mSystems.00331-19)

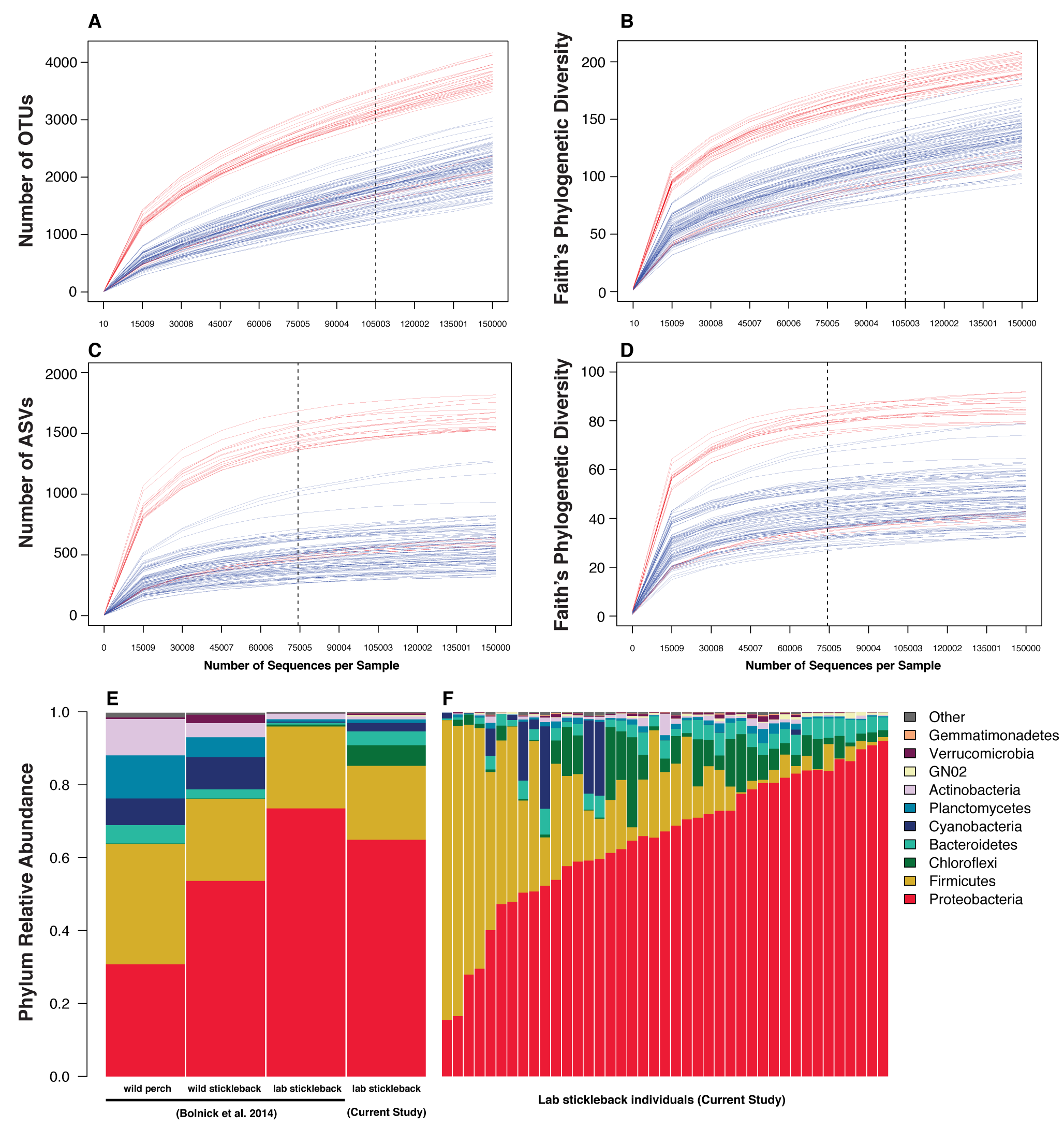

Supplement: FIG S1 [file mSystems.00331-19-sf001.tif]

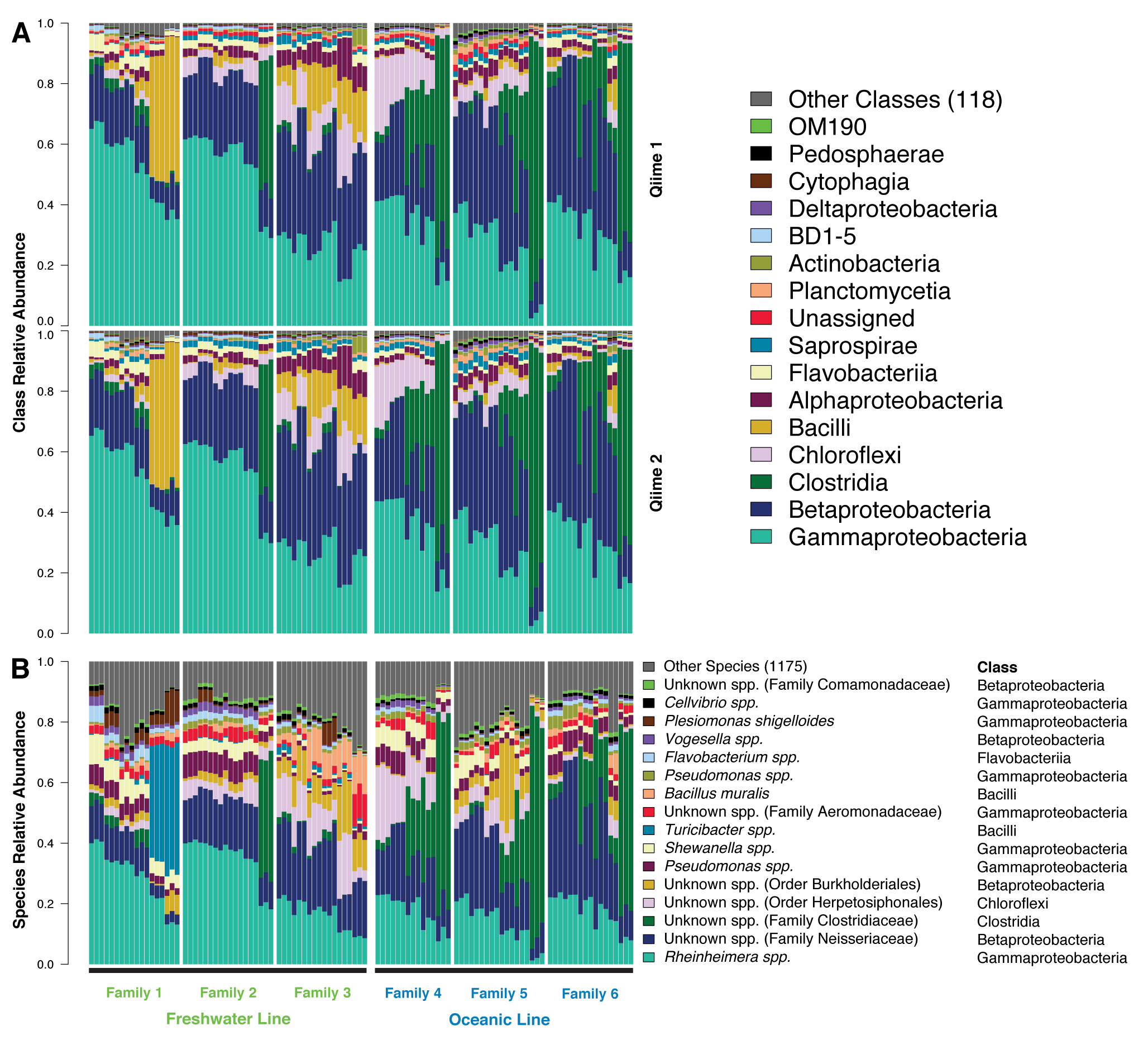

Supplement: FIG S2 [file mSystems.00331-19-sf002.tif]

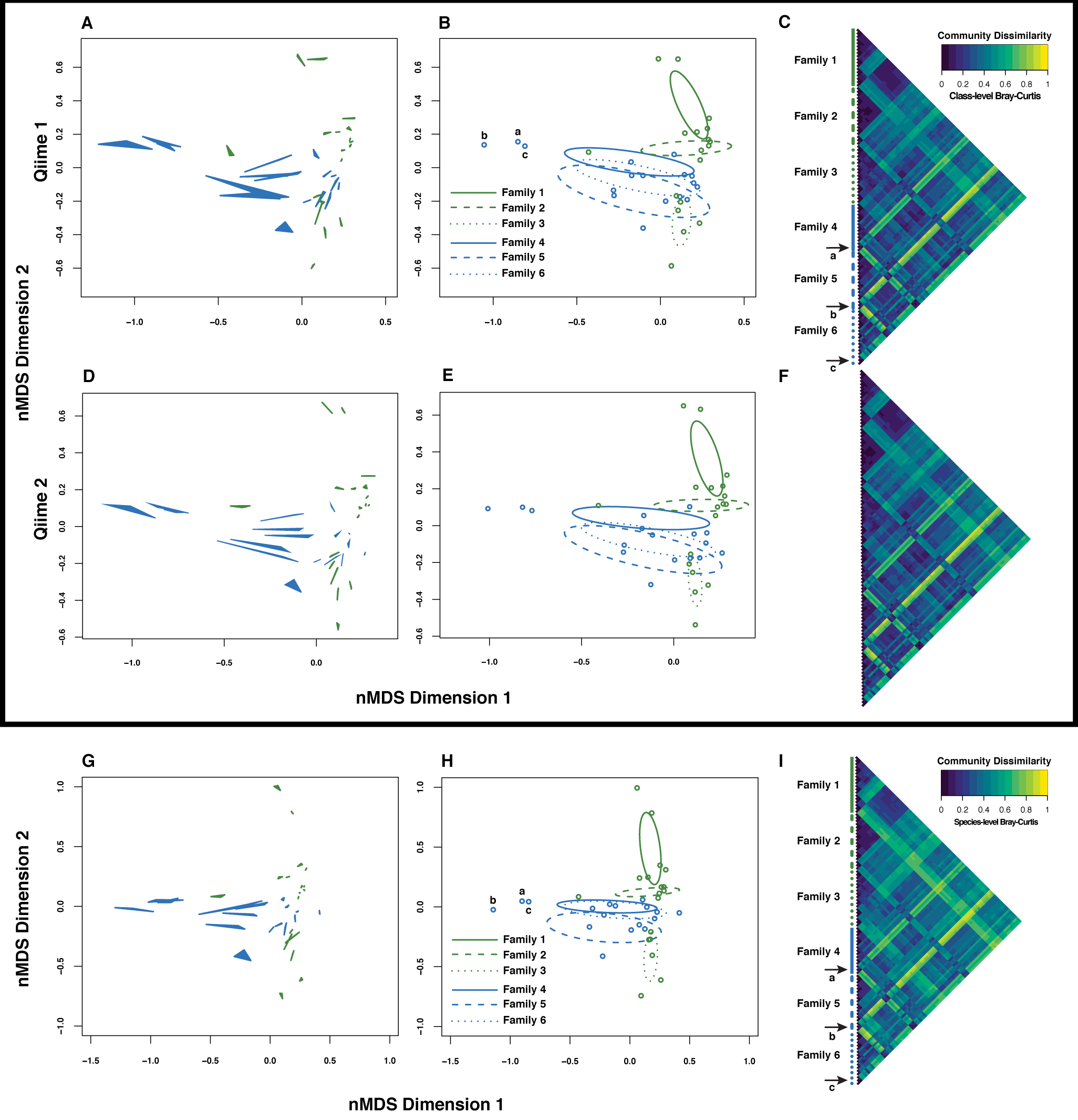

Supplement: FIG S3 [file mSystems.00331-19-sf003.tif]

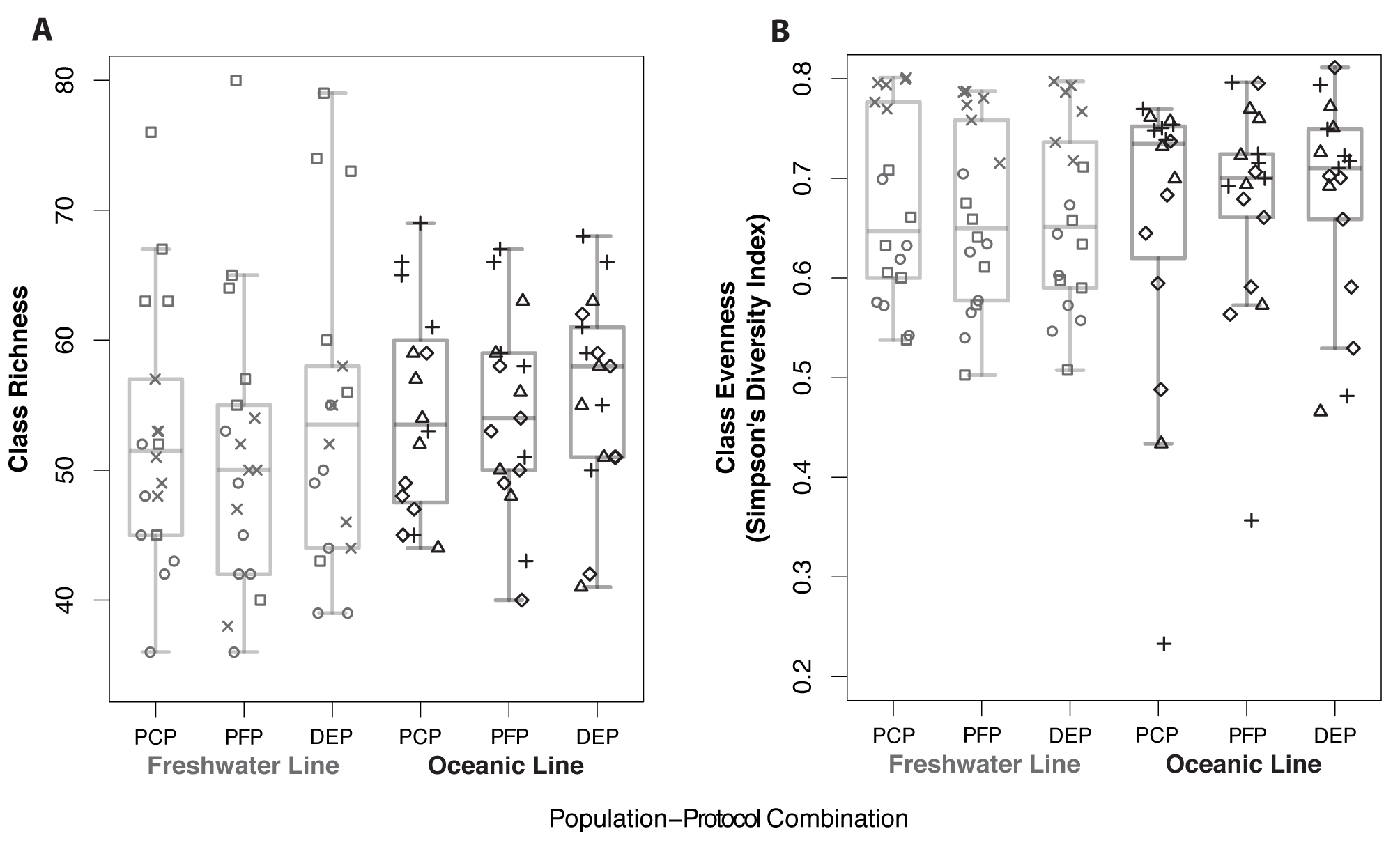

Supplement: FIG S4 [file mSystems.00331-19-sf004.tif]

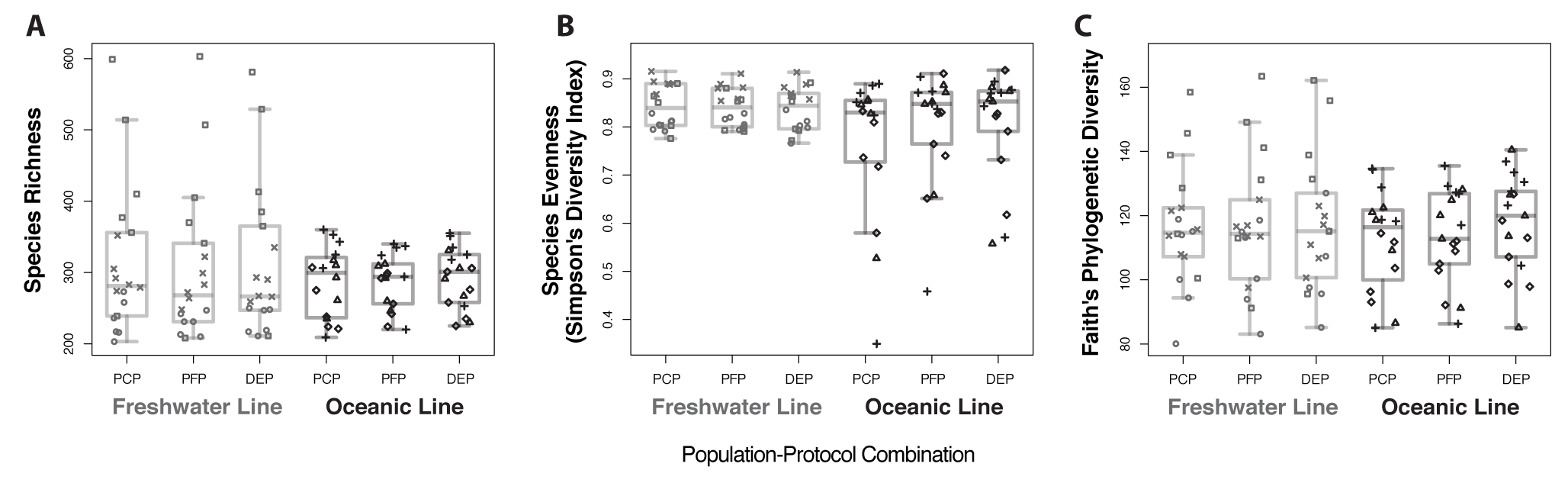

Supplement: FIG S5 [file mSystems.00331-19-sf005.tif]

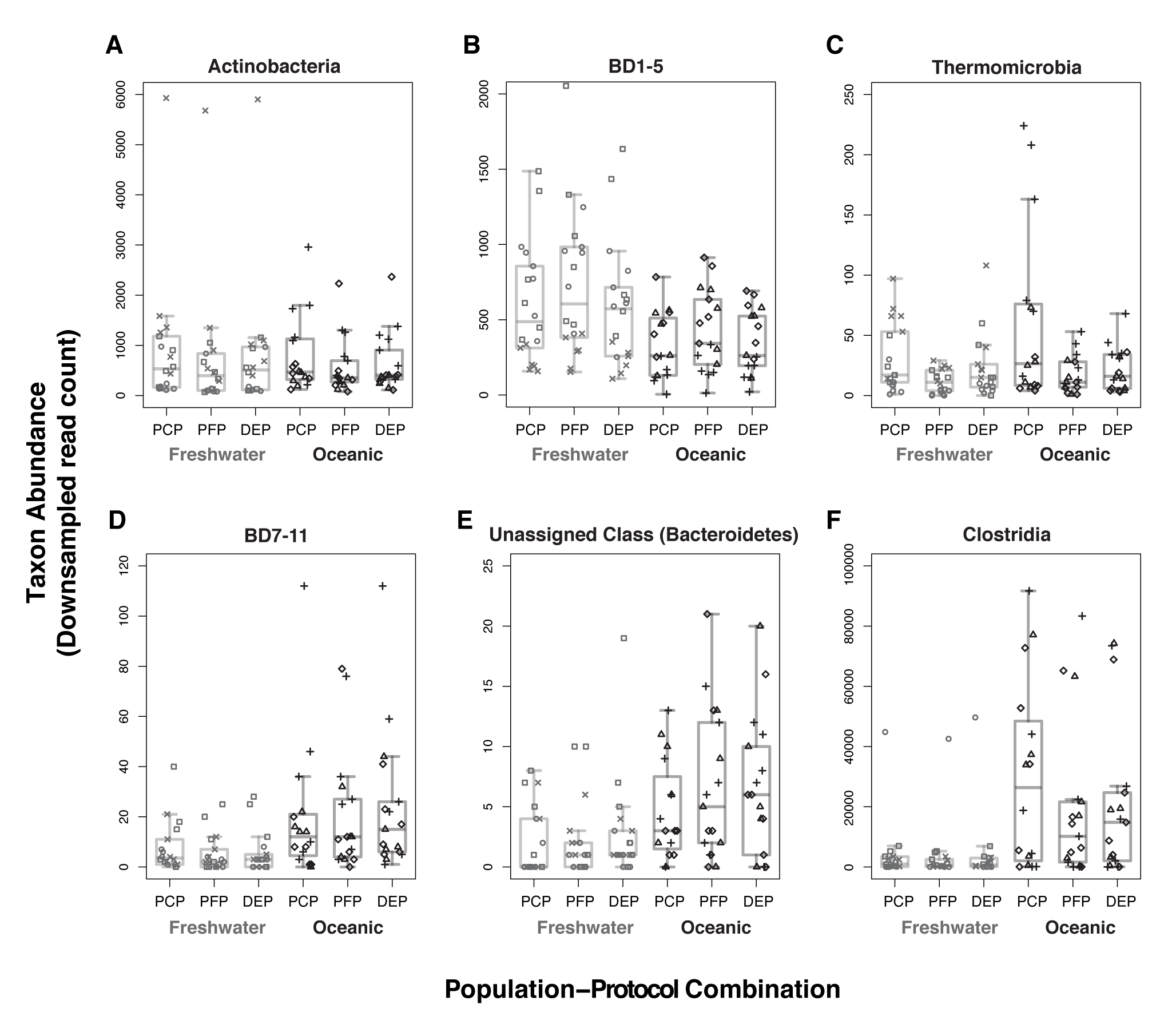

Supplement: FIG S6 [file mSystems.00331-19-sf006.tif]

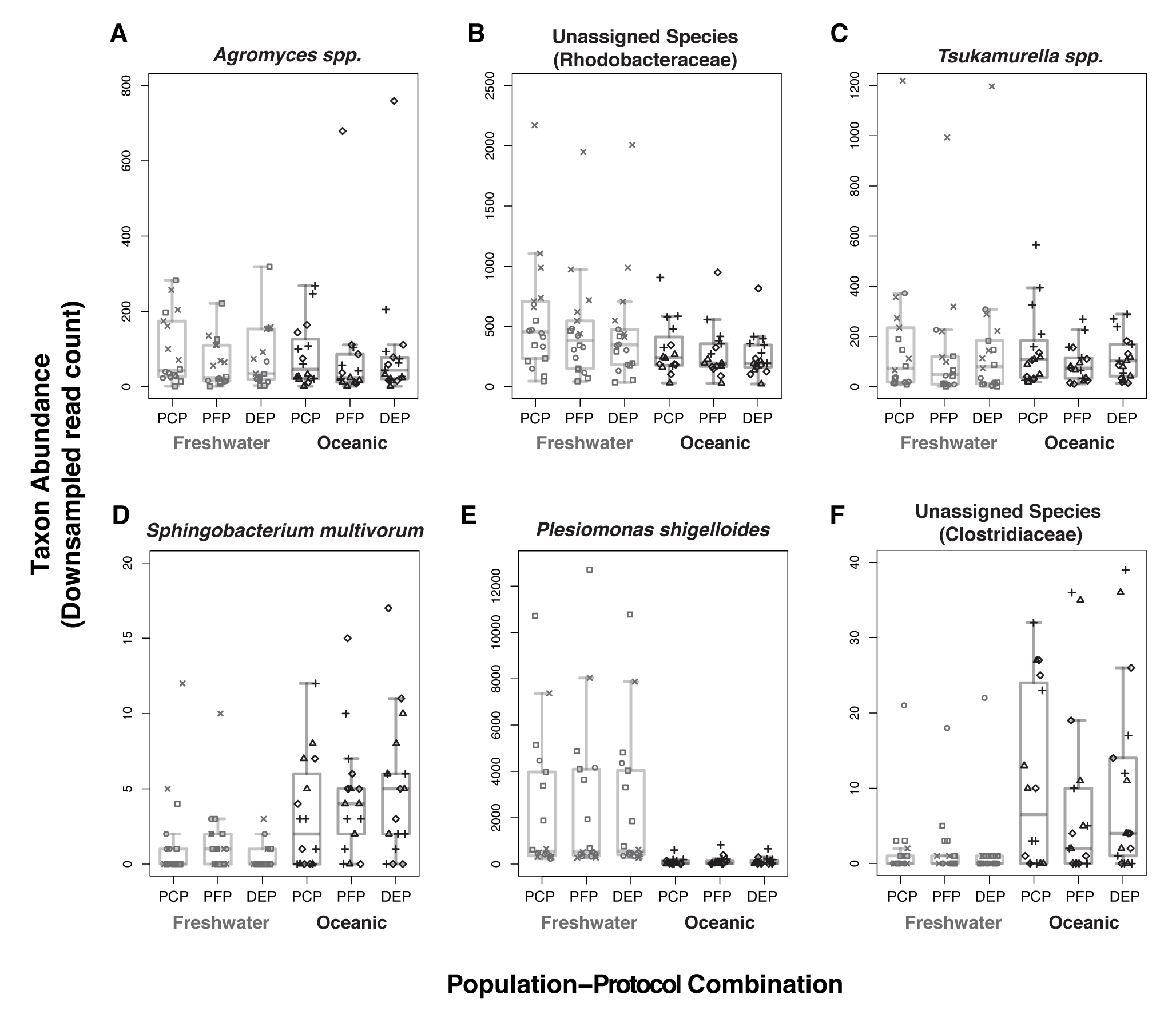

Supplement: FIG S7 [file mSystems.00331-19-sf007.tif]

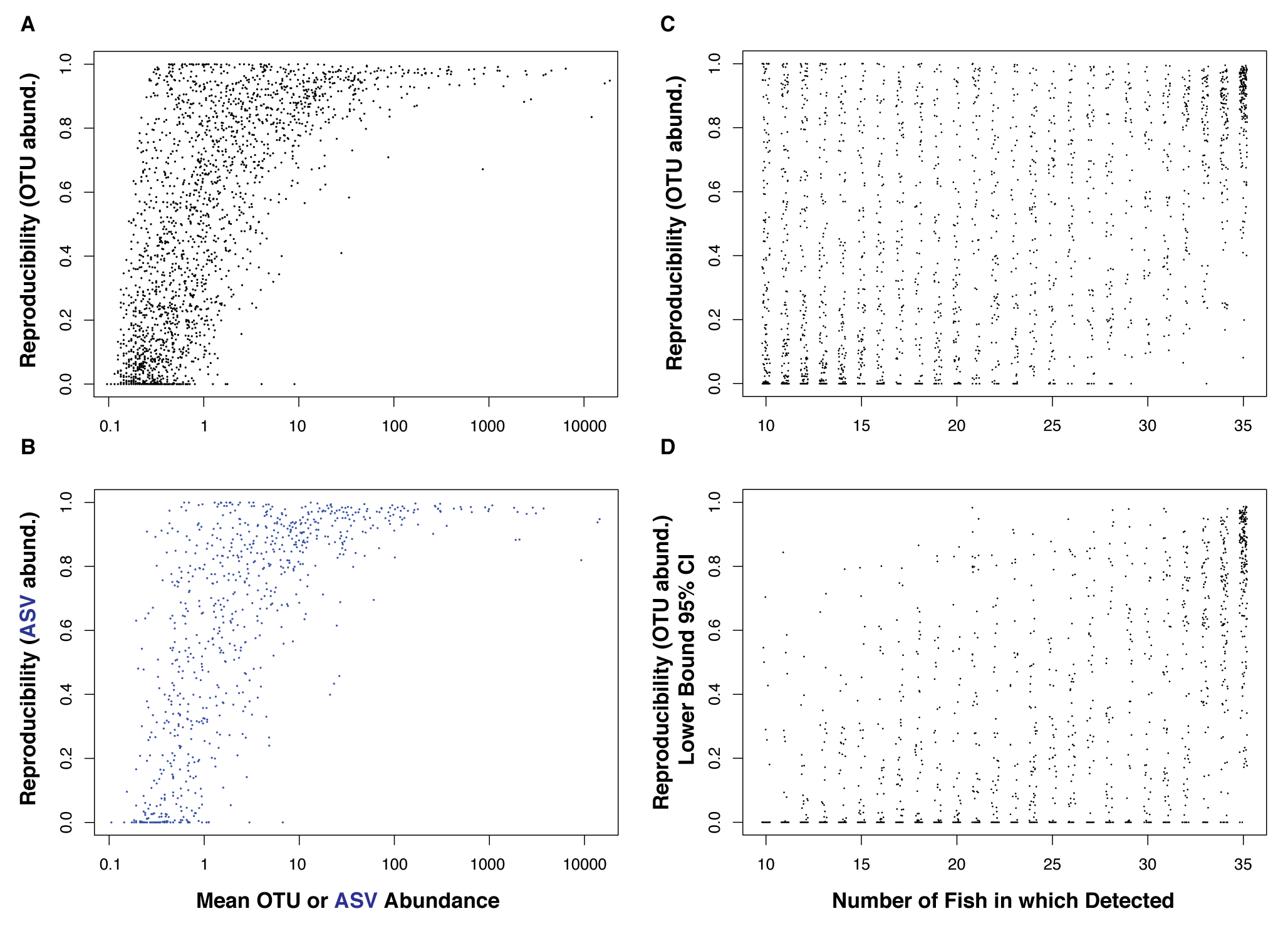

Supplement: FIG S8 [file mSystems.00331-19-sf008.tif]

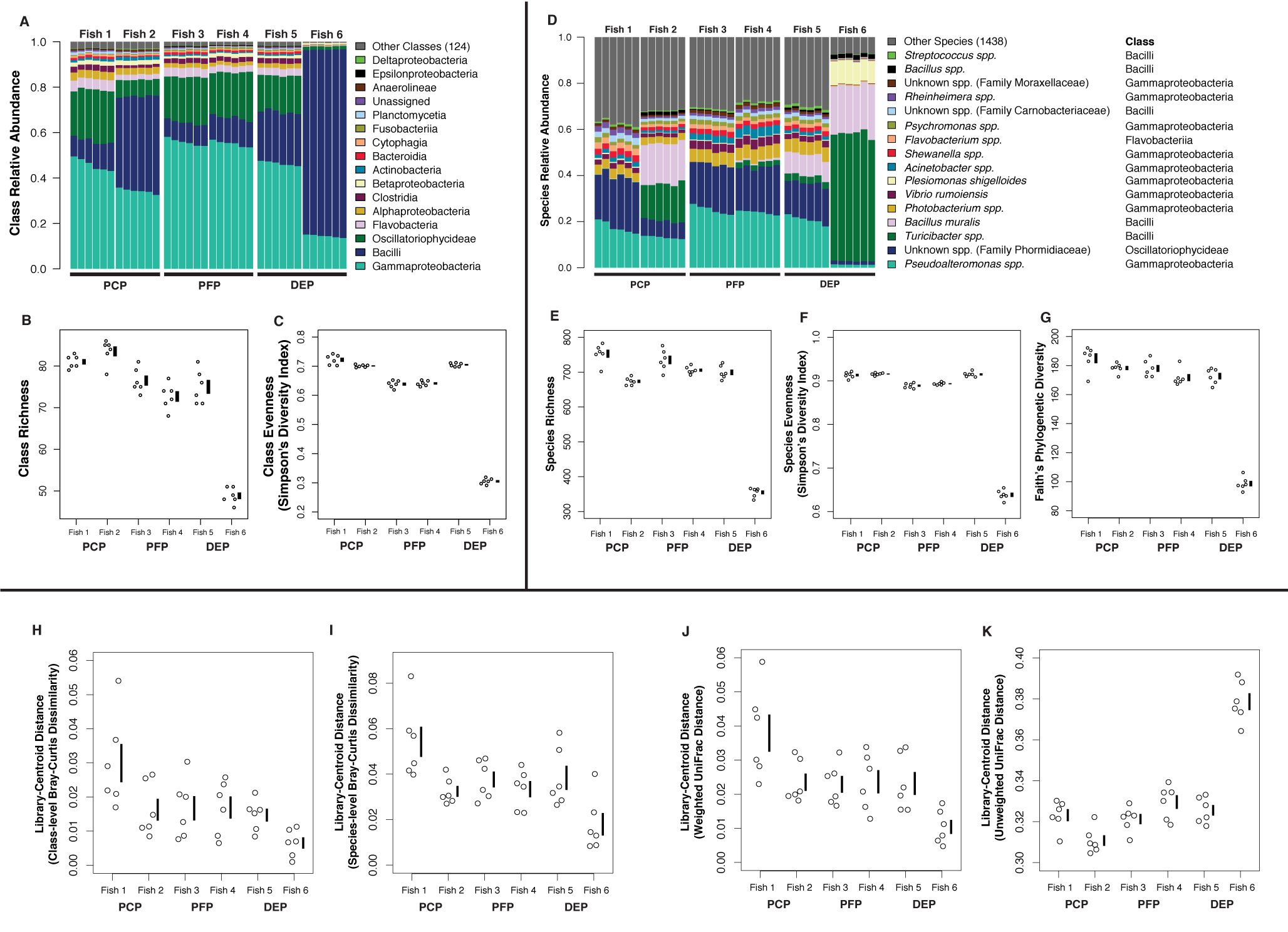

Supplement: FIG S9 [file mSystems.00331-19-sf009.tif]
